# Adolescent survey non-response and later risk of death. A prospective cohort study of 78 609 persons with 11-year follow-up

**DOI:** 10.1186/1471-2458-7-87

**Published:** 2007-05-22

**Authors:** Ville M Mattila, Jari Parkkari, Arja Rimpelä

**Affiliations:** 1 School of Public Health, University of Tampere, 33101 Tampere, Finland; 2 Tampere Research Centre of Sports Medicine, UKK Institute, 33101 Tampere, Finland

## Abstract

**Background:**

Non-response in survey studies is a growing problem and, being usually selective, it leads to under- or overestimation of health outcomes in the follow-up. We followed both respondents and non-respondents by registry linkage to determine whether there is a risk of death, related to non-response at baseline.

**Methods:**

Sample data of biennial surveys to 12-18-year-old Finns in 1979–1997 were linked with national death registry up to 2001. The number of respondents was 62 528 (79.6%) and non-respondents 16 081 (20.4%). The average follow-up was 11.1 years, totalling 876 400 person-years. The risk of death between non-respondents and respondents was estimated by hazard ratios (HR).

**Results:**

The number of deaths per 100 000 person-years were 229 in non-respondents and 447 in respondents (HR 2.0, 95% CI: 1.5–2.6). The hazard ratios of death were for intoxication 3.2 (95% CI: 1.9–5.4), for disease 3.1 (95% CI: 2.2–4.1), for violence-related injury 2.0 (95% CI: 1.5–2.6) and for unintentional injury 1.8 (95% CI: 1.3–2.4) in non-respondents vs. respondents. The association between non-response and death increased with age at baseline, and the increase persisted after the age of 25.

**Conclusion:**

Our study demonstrated significantly increased rates of death among adolescent non-respondents in a follow-up. The highest hazard ratios were seen in disease- and violence-related deaths. The death rate varied between respondents and non-respondents by death type. Increased rates of death persisted beyond the age of 25.

## Background

Non-response has become a growing concern in survey studies. Consequently, extra effort is required to determine its effect on study results. In cross-sectional designs, non-respondents are selected, for example, by poorer health behaviour, health and socio-economic background [1,2]. In cohort studies, health outcomes during follow-up may be related to baseline non-response [3], leading to under- or overestimation of the outcome. Only few studies, with controversial results, have described the association between non-response and death rates [4-6].

No information exists about adolescent non-respondents and their later risk of death from injury and other causes. In Finland, we had the unique opportunity to link a large representative adolescent cohort, including both survey respondents and non-respondents, to national death registry in order to see whether there is a risk of death, and which type of death, related to non-response at baseline.

The aim of this prospective cohort study was to investigate whether adolescent survey non-respondents are selected according to later risk of injury- and disease-related death.

## Methods

### Baseline cohort

The Finnish Adolescent Health and Lifestyle Survey (AHLS) is a nationwide monitoring system of adolescent health and health-related lifestyles, conducted as a postal survey every second year as of 1977 among youths aged 12–18. The main questionnaire, mailed out in the first week of February, is followed by two reminders to non-respondents after three and seven weeks. Sampling, research methods, questions and time of inquiry have been maintained as similar as possible for each year. The samples are drawn from the National Population Register Centre by identifying the participants through a birth-selection procedure. All Finns born on certain days in June, July or August with the mean ages of 12.6, 14.6, 16.6 and 18.6 years at the time of survey are selected. Baseline data for our analyses were collected between 1979 and 1997. Of the baseline cohort of 78 609 persons, 62 528 responded and 16 081 did not (response rate 79.6%). The response rate was lower in males (73.0%) than in females (86.5%) and it was also lower in the older age groups (Table 1).

**Table 1:** Numbers of respondents, non-respondents, and response rates by sex and age.

**Sex and age**	**Number of non-respondents**	**Number of respondents**	**Response rate (%)**
***Males*** Age			
12	1067	4228	79.8
14	2603	8110	75.7
16	3346	9249	73.4
18	3852	7699	66.7
***Females*** Age			
12	704	4340	86.0
14	1300	8893	87.2
16	1491	10 675	87.7
18	1718	9334	84.5
***Total***			79.6

### Follow-up data on death

The outcome variable in the analysis was death after survey response date, obtained from the Finnish Cause-of-Death Statistics, a statutory computerised register covering the entire country [7]. Our baseline cohort (respondents and non-respondents) was linked with the Cause-of-Death Statistics by means of the unique national personal identification number. The follow-up started on day one after survey endpoint April 30 of each data collection year. In the first analysis, the sample was analysed as one entity, the study endpoints being either death or end of follow-up (December 31, 2001). The baseline cohort was followed for an average of 11.1 years, making a total follow-up time of 876 400 person-years. The average follow-up time for respondents was 11.4 years, a total follow-up time of 710 110 person-years and the corresponding figures for non-respondents were 10.3 years and 166 290 person-years. The individual follow-up times varied between 0 and 27 years and thus the ages of the participants ranged from 13 to 41 years at end of follow-up. Association between response status and death was first analysed for the whole baseline cohort, and then separately for each baseline age group (12, 14, 16 and 18 years). All analyses were performed separately for males and females.

In the second analysis, in order to determine if the effect of the response status changed over time, individual follow-up times were divided into three successive phases according to the person's calendar age during follow-up. Hence, three cohorts were formed: 12 to 17, 18 to 24, and 25 and more years. The cut-off points of 18 and 25 years of age were based on the minimum driving age of 18 and the average age of vocational graduation at 25 years in Finland. For the youngest cohort, the follow-up began on April 30 of each data collection year and ended at death before the 18^th ^birthday, or on adolescent's 18th birthday, or on December 31, 2001, whichever came first. The number of adolescents in this phase was 56 005 and the average follow-up period was 2.9 years. All the persons in the youngest cohort, except the deceased, entered the second cohort for whom the follow-up began on the date following the 18^th ^birthday. Those aged 18 at the time of survey entered the second cohort on April 30 of each data collection year. The follow-up of the second cohort ended at death before the 25^th ^birthday, or on adolescents' 25^th ^birthday, or on December 31, 2001. The number of persons in this phase was 78 552 and the average follow-up time was 6.8 years. All the persons in the second cohort, except the deceased, entered the third, oldest cohort that was followed from the 25^th ^birthday until death, or until end of follow-up on December 31, 2001. The number of persons in this phase was 52 805 and the average follow-up period was 5.0 years. Approval was obtained from the Statistics Finland (TK-53-1526-04) for the use of the Cause-of-Death Statistics.

The diagnosis codes in the Official Cause-of-Death Statistics used the 8th (1979–1986), 9th (1987–1995) and 10th (1996–2001) revisions of the International Classification of Diseases (ICD). The Official Cause-of-Death Statistics record the ICD-based external causes of deaths in 54 classes [7] In the present study, deaths by unintentional injury, violence-related injury, intoxication and disease were analysed separately.

### Statistical methods

Cox's regression model was used to study the association between response status and possible death during follow-up. Hazard ratios (HR) were calculated with a 95% confidence interval (95% CI). First, the cohort was divided based on the age at baseline. Next, the cohort was recombined and the follow-up time of each person was divided into three periods according to the person's calendar age during follow-up (14 to 17, 18 to 24 and 25 and more years).

Because of the long AHLS data collection period of 18 years (1979–1997) the baseline data were first divided into two time periods (1979–1988 and 1989–1997) and for each time period separate Cox's regression models were calculated. The hazard ratios for any death in 1979–1988 were 1.9 (95% CI: 1.5–2.4) in non-respondent males and 2.7 (95% CI: 1.7–4.4) in non-respondent females, and for 1989–1997, they were 1.8 (95% CI: 1.4–2.3) and 1.8 (95% CI: 1.1–3.1), respectively. Since no significant time effect was seen, all data were combined for the above noted analyses.

## Results

In the follow-up baseline cohort of 78 609 persons, there were 676 deaths (0.9%), 447 among respondents and 229 among non-respondents (Table 2). Of all deaths, 36.8% were related to violence-related injuries, 30.5% with unintentional injuries and 24.0% with disease (Table 2). The average age at death was 24.3 (range, 13 to 41) years and it was not related to the response status (*p *= 0.46).

**Table 2:** Number and percentage of death types in a cohort of 78 609 Finnish adolescents during a follow-up of 876 400 person-years.

**Death type**	**Number (percentage)**		
	**Non-respondents**	**Respondents**	**All**

Unintentional injury	61 (26.6)	145 (32.4)	206 (30.5)
Violence-related injury	77 (33.6)	172 (38.5)	249 (36.8)
Intoxication	24 (10.4)	35 (7.8)	59 (8.7)
Disease	67 (29.3)	95 (21.2)	162 (24.0)
**Total**	**229 (100)**	**447 (100)**	**676 (100)**

Considering all deaths, non-respondents had an HR of 2.0 (95% CI: 1.5–2.6) compared to respondents (Table 3.). When deaths were analysed separately, the highest HRs among non-respondents were for deaths caused by intoxication (HR 3.2; 95% CI: 1.9–5.4), disease (HR 3.1; 95% CI: 2.3–4.3) and violence-related injuries (HR 2.0; 95% CI: 1.5–2.6).

**Table 3:** Hazard ratios of death by response status in a cohort of 78 609 Finnish adolescents during a follow-up of 876 400 person-years by sex.

**Hazard ratios of death in non-respondents compared to respondents (95% CI)**					
	**Unintentional injury**	**Violence-related injury**	**Intoxication**	**Disease**	**All deaths**

**Males**	**1.4 (1.02–1.9)**	**1.7 (1.3–2.2)**	**2.6 (1.5–5.6)**	**2.9 (1.9–4.3)**	**1.8 (1.5–2.2)**
**Females**	1.5 (0.6–3.8)	1.1 (0.5–2.5)	2.9 (0.8–2.3)	**3.5 (2.1–5.7)**	**2.2 (1.5–3.2)**
**Total**	**1.8 (1.3–2.4)**	**2.0 (1.5–2.6)**	**3.2 (1.9–5.4)**	**3.1 (2.3–4.3)**	**2.3 (1.9–2.6)**

CI = Confidence interval

When the baseline cohort was analysed separately by sex, we noted that non-response in the entire female cohort was associated with an increased risk for all causes of death in the follow-up (2.2 times, 95% CI: 1.5–3.2), while the corresponding risk among non-respondent males was 1.8 (95% CI: 1.5–2.2) (Table 3.). The associative effect of non-response on death increased with age at baseline. Non-response among 16 to 18-year-old males was associated with death caused by disease (HR 2.6; 95% CI: 1.9–4.3), intoxication (HR 2.6; 95% CI: 1.5–5.6) and unintentional injury (HR 1.9; 95% CI: 1.3–2.8). When the female cohort was analysed by age at baseline, non-response was associated with disease-related death at ages 16 to 18 (HR 4.1; 95% CI: 2.3–7.3). (Table 3.)

Finally, when analysing the baseline cohort by persons' age during follow-up, the association between non-response and death was strongest in the oldest age group. Thus, the association persists beyond the age of 25 (Table 4.). Both male and female non-respondents aged 25 or more years showed an increased risk of death from any causes with HR 2.0 (95% CI: 1.5–2.7) and HR 2.6 (95% CI: 1.5–4.8), correspondingly. Non-response was not significantly associated with any category of death before the age of 18.

**Table 4:** Hazard ratios of death by response status in a cohort of 78 609 Finnish adolescents during a follow-up of 876 400 person-years by sex and age at follow-up.

**Hazard ratios of death in non-respondents compared to respondents (95% CI)**					
	**Unintentional injury**	**Violence-related injury**	**Intoxication**	**Disease**	**All deaths**

Age at follow-up					
**Males**					
<18	0.9 (0.4–2.4)	0.9 (0.2–4.3)	-	2.7 (0.6–12.1)	1.1 (0.6–2.3)
18–24	1.3 (0.8–2.0)	**1.8 (1.2–2.8)**	**3.4 (1.3–8.5)**	**2.3 (1.2–4.3)**	**1.7 (1.3–2.2)**
25 or older	**1.9 (1.03–3.5)**	1.6 (1.0–2.6)	**2.4 (1.1–5.1)**	**2.8 (1.5–5.3)**	**2.0 (1.5–2.7)**
**Females**					
<18	0.04 (0-NA)	2.6 (0.5–13.2)	-	0.04 (0-NA)	0.9 (0.2–4.1)
18–24	1.7 (0.6–5.1)	1.1 (0.3–3.0)	**6.4 (1.3–31.6)**	2.3 (1.0–5.2)	**2.0 (1.2–3.4)**
25 or older	2.9 (0.6–14.1)	0.5 (0.1–3.8)	0.0 (0-NA)	**4.4 (2.2–9.0)**	**2.6 (1.5–4.8)**

NA = not applicable

CI = Confidence interval

## Discussion

Our findings demonstrated that adolescent survey non-respondents are selected according to later risk of death. The risk was higher among non-respondents compared to respondents. The excess mortality was particularly associated with disease-related and intoxication deaths, while the difference was smaller for deaths by unintentional and violence-related injury. In this follow-up from adolescence into adulthood, non-respondent males had a significantly increased risk of death by disease, intoxication, violence-related injury and unintentional injury, whereas non-respondent females showed an increased risk of death by disease only. Moreover, the increased risk persisted beyond the age of 25. The association between non-response and risk of death persisted with age better in males than in females.

This study has notable strengths. First, we had the exceptional opportunity to measure the outcome independently of the survey. Second, the study was based on a large, prospective, nationwide sample of adolescents followed over a remarkably long period (876 400 person-years). Third, the coverage and accuracy of the Finnish Cause-of-Death Statistics are known to be excellent [7-9].

The death rates varied between respondents and non-respondents by the type of death. Only few reports describing death rates among non-respondents exist [3,6], but none of them has focused on adolescents. In a study on Taiwanese adults, persons not responding to health surveys had more deaths in a follow-up of two years [4]. A similar finding was published in the United Kingdom, where men not participating in a cardiovascular disease study showed an increased risk of death compared to men participating for a period of three years, after which the difference between the groups disappeared [5]. However, their risk of cardiovascular death did not differ. It should be noted that the follow-up periods of only a few years can be considered relatively short and thus a limitation in these studies.

The degree of a possible bias effect of non-response on adolescent injury and violence survey results has not been previously investigated. Our study showed that, in the follow-up, unintentional injury death rates were 1.8 times, violence-related injury deaths 2.0 and intoxication deaths 3.2 times higher among adolescent non-respondents compared to respondents. The increased risk of injury was especially seen in males. It is evident that non-respondents sustain more injuries, intoxications and violence than respondents. Furthermore, it is remarkable that the death risk associated with non-response persisted after adolescence, even beyond the age of 25. Based on our findings, it seems obvious that the occurrence of injuries and violent deaths as based on cohorts in a survey setting is a clear underestimation.

A previous Finnish study showed that survey non-respondents tend to engage in negative health behaviours, such as smoking, as well as suffer from mental disorders more often than respondents [1]. Moreover, it seems plausible that several risk factors for injuries accumulate in the non-respondent group. Strong evidence exists that use of alcohol, smoking, poor health and low sociodemographic status predict injuries in adolescence [10]. It seems that non-response, particularly in males, is not distributed sporadically but may be regarded as an indicator of a health neglecting lifestyle and a predictor of injury-related death.

Interestingly, non-response was associated with an increased risk for disease death, even more than injury death. Non-response in disease deaths may be a partial consequence of an already existing serious disease or disability at the time of survey. Unfortunately, we had no opportunity to measure this. The survey sample was drawn from the National Population Register Centre through the selection of all Finns born on certain days. In the follow-up, we used the cause-of-death statistics from the survey endpoint, the 30^th ^of April of the survey year and thus, even if death had occurred between the selection of the sample and the response time, it was not registered as death in our analysis.

In our sample, non-response was more common in males and in the older age groups. For example, among 18-year-old males, the response rate was relatively low (67%). A more specific analysis illustrated that while the response rate was 67% in the 18-year-old males and 85% in the 18-year-old females, the injury risk in the non-response group was elevated only in the non-respondent males. In males, the declining response rates from the 1980s to the 1990s did not affect the association between response status and deaths. In females, however, the declining response rates slightly decreased this association.

In summary, the present prospective cohort study is the first to explore death risk among adolescent survey non-respondents. Although death rates vary by death type between respondents and non-respondents, this study shows that the risk of death from any cause (intoxication, violence-related injury, unintentional injury or disease) is increased among adolescent non-respondents and the increased risk persists after the age of 25 years. The predictive strength of non-response, however, seems to persist with age better in females than in males.

## Competing interests

The author(s) declare that they have no competing interests.

## Authors' contributions

AR conceived the study, and participated in its design and coordination and helped to draft the manuscript. JP helped with the design and writing. VM performed the analysis and wrote the first draft. All authors read and approved the final manuscript

## Pre-publication history

The pre-publication history for this paper can be accessed here:


